# CXCL14 Inhibits Colon Cancer Progression by Modulating Tumor Cell Invasion and Immune Microenvironment

**DOI:** 10.3390/cells15100860

**Published:** 2026-05-08

**Authors:** Yinjie Zhang, Siyi Wang, Yuchen Niu, Yanjing Wang, Buyong Ma, Jingjing Li

**Affiliations:** 1 School of Pharmacy, Shanghai Jiao Tong University, 800 Dongchuan Road, Shanghai 200240, China; zhyj@sxu.edu.cn (Y.Z.);; 2 The Key Laboratory of Chemical Biology and Molecular Engineering of Ministry of Education, Modern Research Center for Traditional Chinese Medicine, Shanxi University, Taiyuan 030006, China; 3 Engineering Research Center of Cell and Therapeutic Antibody, Ministry of Education, 800 Dongchuan Road, Shanghai 200240, China

**Keywords:** CXCL14, colorectal carcinoma, epithelial–mesenchymal transition, immuno-microenvironment

## Abstract

CXCL14 is a highly conserved chemokine with potential roles in tumor progression and immune modulation. This study investigates the functional impact of CXCL14 on colon cancer by exploring its effects on tumor cell behavior and the immune microenvironment. We generated stable cell lines overexpressing CXCL14 in mouse MC38 and CT26 cells and human HCT15 colon cancer cells, and used these models to assess tumor growth, invasion, and immune cell infiltration. Our results demonstrate that CXCL14 suppresses colon cancer cell proliferation, migration, and metastasis. In vitro, CXCL14 inhibited the expression of matrix metalloproteinases (MMPs), key regulators of epithelial–mesenchymal transition (EMT), suggesting a role in promoting mesenchymal–epithelial transition (MET). Additionally, in vivo studies using a subcutaneous tumor model showed that CXCL14 not only suppressed tumor growth but also enhanced the infiltration of immune cells, including NK cells, dendritic cells (DCs), and T cells, converting the tumor microenvironment from a “cold” to a “hot” phenotype. RNA sequencing and pathway analyses revealed that CXCL14 regulates the expression of genes associated with angiogenesis, immune response, and cell signaling, particularly through the MAPK pathway. Furthermore, CXCL14’s influence on tumor progression was confirmed in a spleen-to-liver metastasis model, where its overexpression reduced metastatic spread. In conclusion, CXCL14 inhibits colon cancer progression by modulating both tumor cell behavior and the immune landscape, making it a promising candidate for targeted immunotherapy. Our findings highlight CXCL14’s potential to enhance anti-tumor immunity and provide new insights into its therapeutic applications in colon cancer.

## 1. Introduction

CXCL14 is a non-ELR chemokine and highly conserved in mammals (97.4% identity between mice and human proteins), constitutively expressed in normal tissues and involved in immune response, epithelial cell proliferation and migration, angiogenesis [1], and antimicrobial activity [2]. CXCL14 exhibits chemotactic activity for monocytes [3], CD56+ natural killer cells [4].

CXCL14 has the potential to inhibit the motility and migration of tumor cells, including human colonic cancer cells in vitro [5,6] and non-tumor cells, such as trophoblast cells [7] and neural cells [8]. Therefore, CXCL14 may be associated with EMT. Secondly, CXCL14 may play a role in regulating the angiogenesis microenvironment. Research has shown that CXCL14 can inhibit corneal and dental pulp tissue angiogenesis in mice and angiogenesis of human hepatocellular carcinoma in vitro [9,10]. Finally, CXCL14 plays a complex role in colon cancer’s immune microenvironment, possessing anti-tumor effects. CXCL14 can attract and activate natural killer cells (NKs) and macrophages [3,4], enriching them in the tumor microenvironment, and thereby enhancing the regulation of anti-tumor immune responses against these cells. However, in some other cases, CXCL14 exhibits immunosuppressive functions. CXCL14 can indirectly suppress immune responses by modifying the function and/or recruitment of regulatory T cells (T_reg_) [11]. This interference may aid tumor cells in evading attacks from the immune system.

CXCL14 is highly expressed in colon epithelial cells but widely silenced in colon adenocarcinoma, suggesting that this gene plays a tumor suppressor role in colon cancer [12]. Despite these findings, there is still very limited substantive evidence that CXCL14 can inhibit the development of colon cancer cells in vivo. Studying the function and mechanism of CXCL14 will not only enhance our understanding of the etiology and progression of colon cancer but also offer novel insights for the development of immunotherapies for solid tumors. The recruitment of immune cells by CXCL14 may offer new therapeutic targets for combating tumor growth.

Recent studies have highlighted the multifaceted roles of CXCL14 in various biological processes and diseases. For instance, Shapley additive explanation-based feature selection reveals CXCL14 as a key immune-related gene in predicting idiopathic pulmonary fibrosis [13]. This suggests that CXCL14’s role extends beyond cancer to other fibrotic diseases. Additionally, a unique subpopulation of mucosal fibroblasts in colorectal cancer has been identified with tumor-restraining characteristics, further emphasizing the complex interplay between CXCL14 and the tumor microenvironment [14]. Furthermore, CXCL14 has been identified as an essential modulator of TLR9 agonist-induced anti-tumor immune responses [15], highlighting its importance in immune regulation.

In this study, we aim to investigate the functional impact of CXCL14 on colon cancer by exploring its effects on tumor cell behavior and the immune microenvironment. We generated stable cell lines overexpressing CXCL14 in mouse MC38, CT26, and human HCT15 colon cancer cells, and used these models to assess tumor growth, invasion, and immune cell infiltration.

## 2. Materials and Methods

### 2.1. Cell Culture

The mouse colorectal adenocarcinoma cell line MC38 (Cellosaurus CVCL_B288), kindly provided by Dr. Huili Lu’s Lab in the Pharmacy School, Shanghai Jiao Tong University, were cultured in DMEM supplemented with 10% fetal bovine serum (FBS) (SRE0098, Sigma-Aldrich, St. Louis, MO, USA). CT26 (CRL-2638, ATCC, Manassas, VA, USA) and HCT15 cells (CCL-225, ATCC) were cultured in RPMI1640 medium (Adamas life, Shanghai, China, C8013) with 10% FBS. All cells were cultured in a humidified incubator at 37 °C with 5% CO_2_ and 80% humidity. In the proliferation inhibition assay, recombinant human CXCL14 protein, purchased from MCE (Monmouth Junction, NJ, USA; Cat# HY-P72683), was used in the culture medium.

### 2.2. Western Blot (WB)

The CXCL14 antibody, ab137541, was purchased from the Abcam company (Cambridge, UK). The antibodies for cell signal pathway detection were purchased from Cell Signaling Technology, Inc. (Danvers, MA, USA), including anti-Erk1/2 (#4695), anti-phos-Erk1/2 (#4370), anti-Akt (#9272), anti-phos-Akt (#4060), anti-p38 (#9212), and anti-phos-p38 (#4511).

### 2.3. Stable Cell Line Generation

To generate an MC38 cell line inducible expressing CXCL14, we generated a doxycycline (DOX)-inducible lentiviral vector for CXCL14. The open reading frame (ORF) of mouse CXCL14 and an internal ribosome entry site (IRES) were cloned into the pLVX-TetOne-EGFP vector (Addgene, Watertown, MA, USA #171123), generating the pLVX-TetOne-CXCL14 construct. The lentiviral particles were generated according to the Addgene protocol (https://www.addgene.org/protocols/lentivirus-production/ (accessed on 13 February 2023)). The virus particles were used to infect MC38 cells, and 48 h later, puromycin (2 μg/mL) (ST551, Beyotime Biotechnology, Shanghai, China) was added to select infected cells. Stable cell lines were isolated and subsequently induced with 1 µg/mL DOX (cat. no. 24390-14-5, BBI Life Sciences, Shanghai, China) to overexpress CXCL14. The clones with green fluorescence were isolated and further characterized by quantitative PCR to confirm overexpression of CXCL14 (primers are listed in Table S1).

CT26 stable cell line: The ORF of the mouse *cxcl14* gene was cloned into the pcDNA3.1 vector. Clonal cell lines were isolated using 200 µg/mL G418 (Sangon Biotech, cat. no. B540723, Shanghai, China) and verified for overexpression of CXCL14 using quantitative PCR (primers are listed in Table S1).

HCT15 stable cell line: The pHIV-zsgreen plasmid (Addgene #18121) was modified by replacing the *zsgreen* gene with a puromycin resistance gene. The human *CXCL14* ORF was then cloned into the multi-cloning site. The plasmid was transfected into HCT15 cells, and clonal cell lines were selected using 2 μg/mL puromycin. Overexpression of CXCL14 was verified using quantitative PCR (primers are listed in Table S1).

### 2.4. Tumor-Bearing Model

Mice were housed in a specific pathogen-free (SPF) laboratory with a constant temperature of 25 °C and a relative humidity of 40–70%, under a 12 h light/12 h dark cycle.

Subcutaneous tumor-bearing: The subcutaneous tumor-bearing model was performed referred to Wang et al. [16]. A total of 26 seven-week-old male C57BL/6n mice were used for subcutaneous tumorigenesis. The cells were digested with trypsin-EDTA, washed twice with HBSS, resuspended in PBS, and adjusted to a density of 10^7^/mL; 10^6^ cells were injected subcutaneously at the right axilla per mouse. For the tumor proliferation experiment, mice were fed with sterile water (*n* = 8) or 1 mg/mL DOX (*n* = 8) after subcutaneous tumorigenesis, and tumor size and mouse weight were measured every two days until the end of the experiment. For the RNAseq analysis, mice were given free access to 1 mg/mL DOX (*n* = 5) or drinking water control (*n* = 5) when the tumor grew to about 300 mm^3^, and tumor tissue was collected for further detection after 3 days.

The spleen-to-liver metastasis model was performed according to Huot et al. [17]. Ten 7-week-old male C57BL/6n mice (five per group) were generally anesthetized with Zoletil^®^ 50 (tiletamine/zolazepam) (10 mg/kg) and xylazine (5 mg/kg), following a previously described protocol with minor optimizations [18]. Zoletil^®^ 50 was purchased from Virbac (Carros, France) and xylazine (X1251) was from Sigma-Aldrich. A left subcostal incision was made to expose the spleen; 1 × 10^6^ cells in 100 μL PBS were injected into the lower edge of the spleen for each mouse. Subsequently, the body weight was measured every two days. After 21 days, the mice were euthanized, and the proliferation of tumors in the spleen and liver was observed.

At the end of the experiment, the mice were euthanized by CO_2_ asphyxiation at a volume displacement rate of 50%, and death was confirmed by cervical dislocation.

### 2.5. RNA Sequencing (RNA-seq)

RNA-seq experiments were performed by Beijing Geneplus Technology Co., Ltd. In an in vitro experiment, cell samples included the control and CXCL14 overexpression groups, with two biological replicates per group. In vivo tumor-bearing samples were set up with the control and overexpressing cell groups, each containing four biological replicates. Each sample was sequenced at a depth of 10G.

### 2.6. Quantitative PCR (qPCR)

Total RNA was extracted using Trizol (Invitrogen, Carlsbad, CA, USA 15596026) and reverse-transcribed to cDNA (Novozymes, Bagsvaerd, Denmark, R302-01). Using Sybrgreen quantitative PCR reagents (TAKARA BIO INC., Shanghai, China, RR820Q), gene expression was measured by the ΔΔCt method. Quantitative PCR primers are shown in Table S1.

### 2.7. Invasion Assay

Invasion assay was performed with reference to a previous study [19]. Briefly, 10^5^ cells were used in the assay. The transwell instrument was cultured at 37 °C for 24 h. Five random fields of view at 10× magnification were selected, and the number of cells that have invaded the lower compartment of the invasion chamber is counted.

### 2.8. Flow Cytometry

Tumor tissues were chopped into small pieces (1 mm^3^), added to 5 volumes of digestion solution (1% collagenase II (1710-1015, Gibco, Waltham, MA, USA) + 100 U/mL Hyalurondase (Cat# H3506, Sigma-Aldrich, St. Louis, MO, USA) + 0.1% DNAseI (C600650, BBI Life Sciences, Shanghai, China) + DMEM/F12), and the mixture was incubated at 37 °C for 1 h, with gentle mixing every 15 min. The cells were filtered through a cell strainer to obtain a single-cell suspension. The cells were washed twice with PBS and blocked with PBS containing 1% FBS for 10 min; cells were adjusted to 1 × 10^7^/mL, and 0.1 mL was transferred to an EP tube. Fc receptors were blocked by incubating with 0.5–1 μg of purified monoclonal antibody against mouse CD16/32 (E-AB-F0997A, Elabscience Biotechnology Co., Ltd., Wuhan, China) at room temperature for 10 min. T lymphocyte subpopulations were stained using antibodies against CD3-pc5.5 (45-0031-82, eBioscience, Thermo Fisher Scientific, San Diego, CA, USA), CD4-FITC (11-0041-82, eBioscience), and CD8-APC (eBioscience, 17-0081-82). For macrophage staining, double staining was performed using F4/80-APC (eBioscience, 17-4801-82) and CD11b-PC7 (eBioscience, 25-0112-82), fixed with 1% paraformaldehyde for 10 min in RT, and the cells were then analyzed using a flow cytometer (CytoFLEX, Beckman, Brea, CA, USA).

### 2.9. Data Mining and Analysis

**Figure 1:**
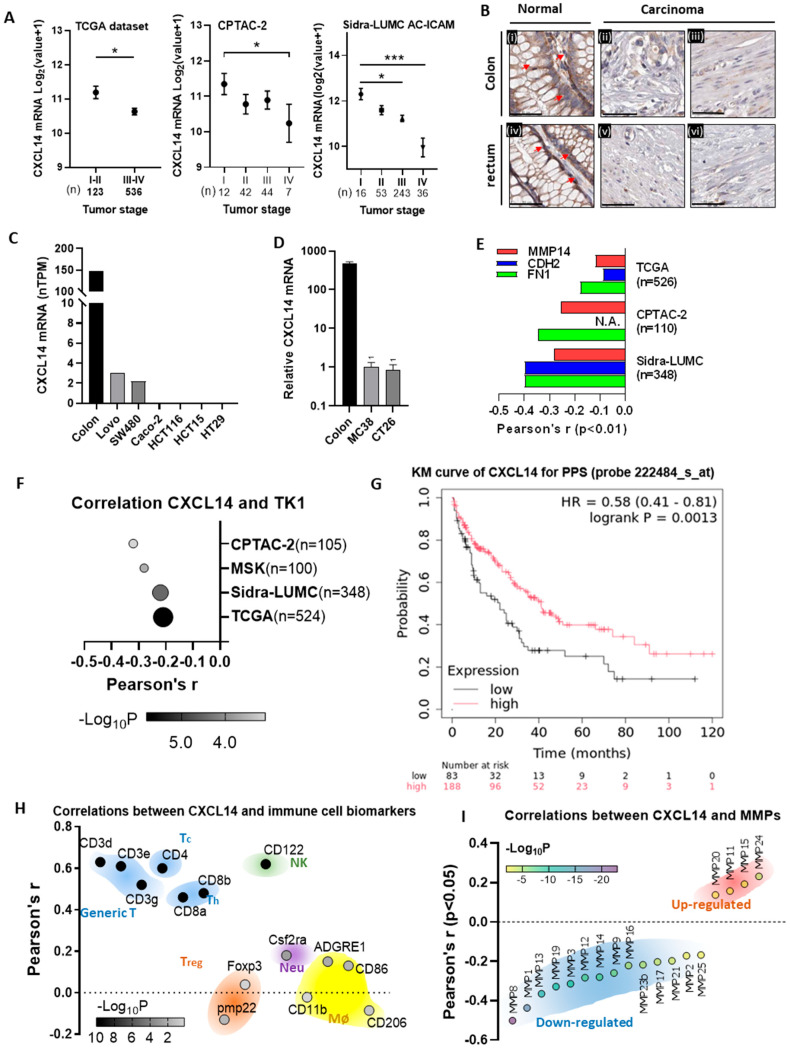
*CXCL14* silencing is associated with adverse prognosis of CRC. (**A**) CRC datasets show CXCL14 downregulation in cancer development and metastasis (mean + SEM). * *p* < 0.05, *** *p* < 0.001. (**B**) The ProteinAtlas database demonstrates that CXCL14 is highly expressed in normal colon ((**i**), PID3753) and rectal ((**iv**), PID:3243) gland cells (arrow), but significantly reduced in colonic adenocarcinoma tissue ((**ii**), PID:2002; (**iii**), PID:1958) and rectal adenocarcinoma ((**v**), PID:1424; (**vi**), PID:1416). The bar represents 50 μm. (**C**) CXCL14 is silenced in human colon cancer cell lines (data from GTEx database) compared to normal tissue (data from ProteinAtlas database). (**D**) Mouse colon tissue and cell line qPCR results show very low CXCL14 in MC38 and CT26 cells. (**E**) Clinical datasets reveal CXCL14 negatively correlates with EMT markers in colon cancer (*p* < 0.01). (**F**) CXCL14 exhibits a general negative correlation with the tumor cell proliferation marker TK1 in the four CRC datasets. (**G**) Kaplan–Meier curve of post-progression survival (PPS) for colon cancer patients stratified by CXCL14 expression (probe 222484_s_at). (**H**) CXCL14 strongly correlates with T cell (0.4 < r < 0.7) and NK cell markers (r > 0.6) in GTEx colon tissue datasets, but weakly or not at all with Treg cells, neutrophils, and macrophages (r < 0.2). (**I**) Analysis of the correlation between CXCL14 expression levels and the expression levels of MMP family genes in the Sidra-LUMC dataset.

The cBioportal database was utilized for the analysis and retrieval of expression profile data regarding colon cancer. The “Co-expression” feature aids in the retrieval of the correlation between the expression levels of other genes and the *CXCL14* gene in the TCGA (PanCancer Atlas [20]), Sidra-LUMC AC-ICAM (Nat Med 2023 [21]), CPTAC-2 Prospective (Cell 2019 [22]) and MSK (Nature Medicine 2022) datasets. The CXCL14 mRNA data for the normal human colon tissue (column Colon) was from the ProteinAtlas database, and the expression level of other human CRC cell lines come from the GTEx database (Figure 1C). The Gene Ontology (GO) and Kyoto Encyclopedia of Genes and Genomes (KEGG) enrichment analysis of RNA-seq data was carried out using the online analysis system provided by JYJCloud (https://www.jyjcloud.com/), and the self-built gene set GO enrichment analysis was conducted on the official website of The Gene Ontology Resource [23].

### 2.10. Statistical Analysis

Correlation analysis and *t*-test were performed using IBM SPSS software (V25). The Pearson correlation coefficient was used to represent the correlation of gene expression levels. A *t*-test was performed with the two-tail model.

## 3. Results

### 3.1. Silencing of the CXCL14 Gene Is Associated with Poor Prognosis in Clinical Cases

We analyzed three major colon cancer datasets (TCGA, CPTAC, and Sidra-LUMC) using the cbioportal database. The results revealed a negative correlation between CXCL14 expression and the stage of colon cancer (Figure 1A), consistent with previous studies on the epigenetic silencing of CXCL14 [12]. The pathological sections of clinical samples indicated that CXCL14 is silenced in tissues of colon cancer and rectal cancer (Figure 1B). Furthermore, common human and mouse CRC cell lines also exhibit almost complete silencing of CXCL14 (Figure 1C,D). Similarly, in the three datasets mentioned above, CXCL14 exhibits a significant negative correlation with several crucial EMT-related genes, MMP14, CDH2, and FN1 (*p* < 0.01, Figure 1E and Figure S1) [24]. The three databases mentioned above, as well as a rectal cancer dataset (MSK, Nature Medicine 2022 [25]), all demonstrate a negative correlation between CXCL14 expression and the tumor proliferation marker TK1 in colon cancer cells (*p* < 0.001), indicating that CXCL14 is inversely associated with tumor proliferation (Figure 1F and Figure S2).

To further evaluate the prognostic value of CXCL14 in colon cancer, we performed Kaplan–Meier survival analyses using the KM Plotter database [26], focusing on two independent Affymetrix probes (218002_s_at and 222484_s_at) for three survival endpoints: relapse-free survival (RFS), overall survival (OS), and post-progression survival (PPS). As shown in Figure 1G, high expression of CXCL14 detected by the probe 222484_s_at was significantly associated with prolonged PPS (log-rank *p* = 0.0013). In contrast, no significant or weak associations were observed for RFS or OS using the same probe (*p* = 0.20 and *p* = 0.028, respectively; the latter did not reach the predefined significance threshold after correction for multiple testing). Consistent but slightly weaker results were obtained with the other probe (218002_s_at), which are provided in Figure S3. These findings further support a specific role of CXCL14 in improving survival after disease progression, beyond its effects on primary tumor growth or recurrence.

To explore the correlation between CXCL14 and immune cells, we utilized the GTEx database to analyze the relationship between CXCL14 and various immune cell markers in colon tissue. We observed a strong correlation between the expression level of CXCL14 and T cell markers (CD3, CD4, CD8) in the tissue (r > 0.4, *p* < 0.001), as well as a robust correlation with the NK cell marker CD122 (r > 0.6, *p* < 0.001). However, the correlation between CXCL14 and T_reg_ cells, neutrophils, and macrophages is either non-significant or weak (Figure 1H and Figure S4). This finding suggests that CXCL14 may play a role in tumor immune surveillance by recruiting T cells and NK cells to the colon tissue, aligning with previous research [4].

We also discovered in the Sidra-LUMC tumor dataset that CXCL14 expression exhibits a significant negative correlation with most matrix metalloproteinases (MMP1, 2, 3, 8, 9, 12, 13, 14, 16, 17, 23b, 25) (Figure 1F), indicating that CXCL14 may regulate tumor cell metastasis by controlling the expression of matrix metalloproteinases (MMPs). These findings suggested that CXCL14 is strongly associated with tumor suppressor phenotypes.

### 3.2. CXCL14 Inhibits the Proliferation of Colon Cancer Cells In Vitro and in Subcutaneous Tumors

The CXCL14 gene is silenced in almost all malignant colorectal cancers, and this silencing is primarily epigenetic. Therefore, the gene is inherently inactivated in colorectal cancer cells, so reducing CXCL14 expression itself makes no sense. That is why we did not design experiments to knock out or knock down CXCL14. For data on the silencing of CXCL14 expression, please refer to Figure 1C,D and references [5,6]. Therefore, in this study, we only examined the conditions where the tumor overexpresses CXCL14.

**Figure 2:**
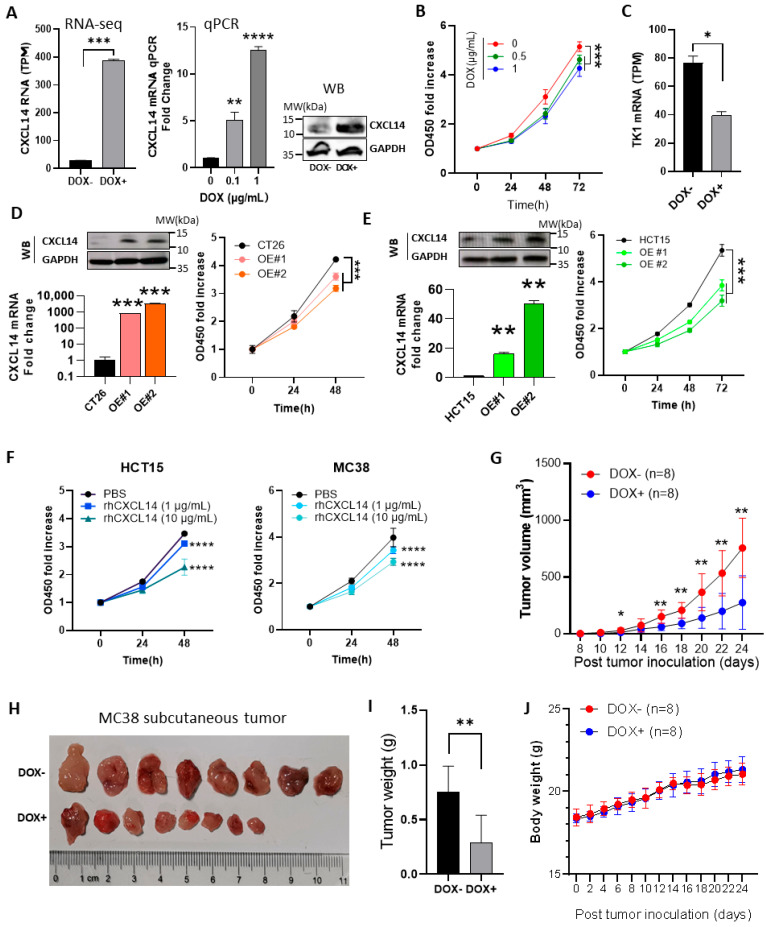
Overexpression of CXCL14 inhibits proliferation of colon cancer cells. (**A**) RNA-seq data (*n* = 2), q-PCR (*n* = 3) and WB result of CXCL14 expression in the non-induced and DOX-induced MC38-TetOn-CXCL14 cell line. Data represent the mean ± SD. ** *p* < 0.01, *** *p* < 0.001, **** *p* < 0.0001, *t*-test. (**B**) Proliferation curves of control and 0.5 and 1 μg/mL DOX-induced MC38 cell (*n* = 6). Data represent the mean ± SD. *** *p* < 0.001, one-way ANOVA. (**C**) RNA-seq data of TK1 mRNA of control and *CXCL14*-overexpressed MC38 cell (*n* = 2). Data represent the mean ± SD. * *p* < 0.05, *t*-test. (**D**) WB detection, qPCR data of *CXCL14* expression (left) and proliferation curves of parental and CXCL14-overexpressed CT26 cells. Data represent the mean ± SD. *** *p* < 0.001, *t*-test and one-way ANOVA. (**E**) WB assay and qPCR data of *CXCL14* expression (left) and proliferation curves of parental and *CXCL14*-overexpressed HCT15 cells. Data represent the mean ± SD. ** *p* < 0.01, *** *p* < 0.001, *t*-test and one-way ANOVA. (**F**) The proliferation inhibition effect of recombinant human CXCL14 (rhCXCL14) on colon cancer cell line in vitro. **** *p* < 0.0001, one-way ANOVA. (**G**) The tumor growth curves of DOX-induced and non-induced groups of C57bl/6n mice with subcutaneous MC38-plvx-TetOn-CXCL14 cells. The tumor volume of the DOX-induced group was significantly lower than that of the non-induced group (*n* = 8), * *p* < 0.05, ** *p* < 0.01, *t*-test. (**H**) Appearance of dissected subcutaneous tumor tissue (*n* = 8 for control and DOX+ group). (**I**) The tumor tissue was excised from the subcutaneous tissue and weighed. The tumor weight of the DOX-induced group was significantly lower than that of the non-induced group (*n* = 8), ** *p* < 0.01, *t*-test. (**J**) The body weight curves of non-induced and DOX-induced mice (*n* = 8), no significant differences by *t*-test.

A CXCL14-induced expression cell line, named MC38-TetOn-CXCL14, was generated [27] (Figure S5). Treatment with 1 μg/mL of DOX induced an increase in *CXCL14* mRNA levels (14-fold by qPCR) and protein levels (by WB) (Figure 2A). CXCL14 overexpression results in a notable decrease in cell proliferation rate (Figure 2B). To rule out any potential influence of DOX on cell proliferation rate, we evaluated its effect on the parental MC38 cells. The results showed no effect at a concentration of 1 μg/mL (Figure S6). Cells overexpressing CXCL14 demonstrated a 50% decrease in the RNA level of Thymidine kinase 1 (TK1) (Figure 2C), a well-established proliferation marker [28]. The overexpression of mouse and human *CXCL14* genes in CT26 and HCT15 cells has also been observed to inhibit cell proliferation. The intensity of this inhibitory effect is proportional to the expression level of CXCL14 (Figure 2D,E).

To further validate the direct anti-proliferative effect of CXCL14 and to exclude potential off-target effects associated with stable overexpression, we treated human HCT15 and mouse MC38 colon cancer cells with recombinant human CXCL14 (rhCXCL14) protein. As shown in Figure 2F, rhCXCL14 treatment significantly inhibited the proliferation of both cell lines. Notably, a clear dose-dependent effect was observed at concentrations of 1 µg/mL and 10 µg/mL. These results are consistent with our observations from CXCL14 overexpression experiments (Figure 2B,D,E), confirming that CXCL14 directly suppresses colon cancer cell growth. The concordance between overexpression and recombinant protein treatment further strengthens the conclusion that CXCL14 exerts cell-autonomous anti-proliferative effects on colon cancer cells.

MC38-TetOn-CXCL14 cells was used to study the effect of CXCL14 on subcutaneous tumors. Sixteen C57BL/6n mice were subcutaneously inoculated with cells and separated into the DOX+ and DOX− groups. The former group was fed with 1 mg/mL DOX in drinking water. The tumor growth rate of the DOX+ group was significantly reduced (*p* < 0.001, Figure 2G). At the end of the experiment, the tumor size and weight in the CXCL14 overexpression group was significantly smaller than that in the control group (Figure 2H,I). The largest tumor measured 1198 mm^3^ in volume, with dimensions of 16 mm in length and 12 mm in width. Throughout the entire experimental period, there was no significant difference in body weight between the two groups (Figure 2J).

### 3.3. Transcriptome Analysis of Colon Cancer Cells with CXCL14 Overexpression

Monolayer MC38-TetOn-CXCL14 cells were induced to overexpress CXCL14 with 1 μg/mL DOX for 3 days and the total RNA was collected for RNA sequencing analysis by Geneplus, Beijing, China (see Data Availability Statement). Each sample had two biological replicates: DOX+ cells and DOX− cells.

**Figure 3:**
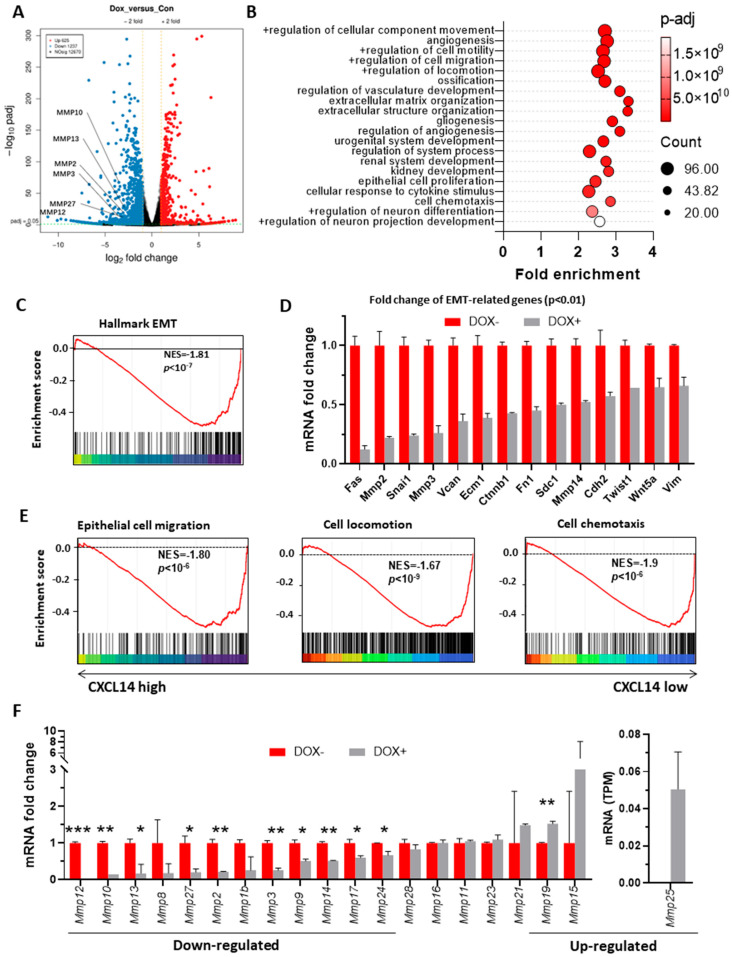
RNA-seq analysis of the regulatory role of CXCL14 overexpression on the transcriptome of MC38 cells. (**A**) The volcanic plot displayed that the induction of CXCL14 expression led to a significant upregulation of 625 genes and downregulation of 1237 genes (*p*-adj *<* 0.05) in MC38 cells, representing approximately 15% of the total genes. (**B**) The Gene Ontology (GO) analysis of differentially expressed genes (DEGs) between the non-induced and DOX-induced MC38-TetOn-CXCL14 cell line. (**C**) GSEA showed that CXCL14 significantly suppressed cell EMT signaling. Dot size indicates the number of genes counted in each term. (**D**) RNA-seq data of EMT-related genes expression of MC38-TetOn-CXCL14 cell line prior or post DOX induction (all *p*-values *<* 0.01). (**E**) The GSEA analysis of the impacts of CXCL14 overexpression on MC38 cell migration, locomotion and chemotaxis. (**F**) RNA-seq data of MMP family genes of non-induced and DOX-induced MC38-TetOn-CXCL14 cell line (*n* = 2). Here only genes with non-zero values are displayed. family. * *p* < 0.05, ** *p* < 0.01, *** *p* < 0.001.

The volcanic plot showed that 625 genes were upregulated and 1237 genes were downregulated after DOX-induced CXCL14 expression (Figure 3A). GO enrichment analysis revealed enrichment in cell motility, migration, angiogenesis, and extracellular matrix-related genes (Figure 3B; fold enrichment > 2, *p* < 10^−9^). GSEA indicated that CXCL14 overexpression mainly affected the EMT negative regulation pathway (Figure 3C; NES = −1.81) and downregulated numerous EMT-related genes (Figure 3D, *p* < 0.01). CXCL14 also inhibited epithelial cell migration, motility, chemotaxis (Figure 3E), and MMPs family gene expression (Figure 3F). These findings were supported by the colon cancer Sidra-LUMC dataset (Figure 1I).

### 3.4. CXCL14 Overexpression Inhibits the Invasion of Colon Cancer Cells

**Figure 4:**
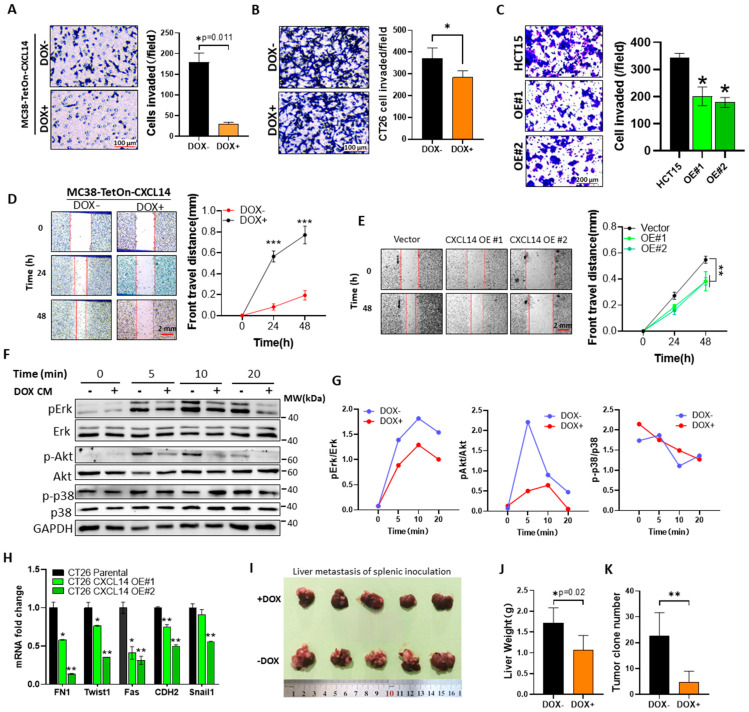
Overexpression of CXCL14 inhibits invasion of colon cancer cells. (**A**) The invasion assay of non-induced and DOX-induced CXCL14-overexpressed MC38 cells (*n* = 3). Data represent the mean ± SD. * *p* = 0.011, *t*-test. (**B**) The invasion assay demonstrates that overexpression of CXCL14 in CT26 cells impairs their invasion capability (*n* = 3), * *p* < 0.05, *t*-test. (**C**) The invasion assay has revealed that the restored expression of CXCL14 in HCT15 cells effectively suppresses their invasion capacity. * *p* <0.01, *t*-test. (**D**) The wound repair assay of control and 1 μg/mL DOX-induced MC38 cell (*n* = 10). The red lines indicate the leading edge of the migrating cells. Data represent the mean ± SD. *** *p* < 0.001, *t*-test. (**E**) The wound repair assay of parental and CXCL14-overexpressed-HCT15 cell lines (*n* = 6). Data represent the mean ± SD. ** *p* < 0.01, one-way ANOVA. (**F**) WB assay of the effect of CXCL14-conditioned medium on the phosphorylation of Erk, Akt, and p38 proteins in MC38 cells. (**G**) The grayscale quantification results of the WB bands in (**F**). the data represents the consistent outcomes from multiple replicates of the experiment. (**H**) qPCR data of EMT-related genes in parental and CXCL14-overexpressed CT26 cells (*n* = 2), * *p* < 0.05, ** *p* <0.01, *t*-test. (**I**) The appearance of the liver 21 days after spleen inoculation with MC38. (**J**) The liver weight data, *n* = 5, * *p* = 0.02. (**K**) Number of metastatic tumor foci on the liver surface. ** *p* < 0.01, *n* = 5.

CXCL14 significantly reduces the invasion capability of MC38 cells when induced by DOX, as shown in Figure 4A, with no inhibitory effect observed from DOX alone. Similar results were seen in transient transfection experiments (Figure S7) and in CT26 (Figure 4B) and HCT15 cells (Figure 4C), indicating a broad impact on cell invasion. Scratch tests (Figure 4D,E) also showed reduced motility in MC38 and HCT15 cells expressing CXCL14.

In MC38 cells treated with CXCL14-conditioned medium, phosphorylation of Erk and Akt was significantly inhibited, as illustrated in Figure 4F,G, while p38 phosphorylation was unaffected. qPCR analysis in CT26 cells with CXCL14 overexpression (Figure 4H) revealed suppressive effects on EMT-related gene expression. The same EMT-related gene profiles were also found in CXCL14-overexpressed HCT15 cells (Figure S8).

Furthermore, a spleen tumor-bearing liver metastasis model (Figure 4I–K) demonstrated that CXCL14 overexpression significantly decreased liver metastasis, as evidenced by lower liver weight and fewer metastatic foci. These findings suggest a potential therapeutic role for CXCL14 in reducing metastasis. At the end of the experiment, the maximum liver weight of the mice did not exceed 2.5 g, so their tumor burden did not exceed 10% of the body weight, which met the requirements of animal ethics.

### 3.5. CXCL14 Regulates Tumor Immune Microenvironment

**Figure 5:**
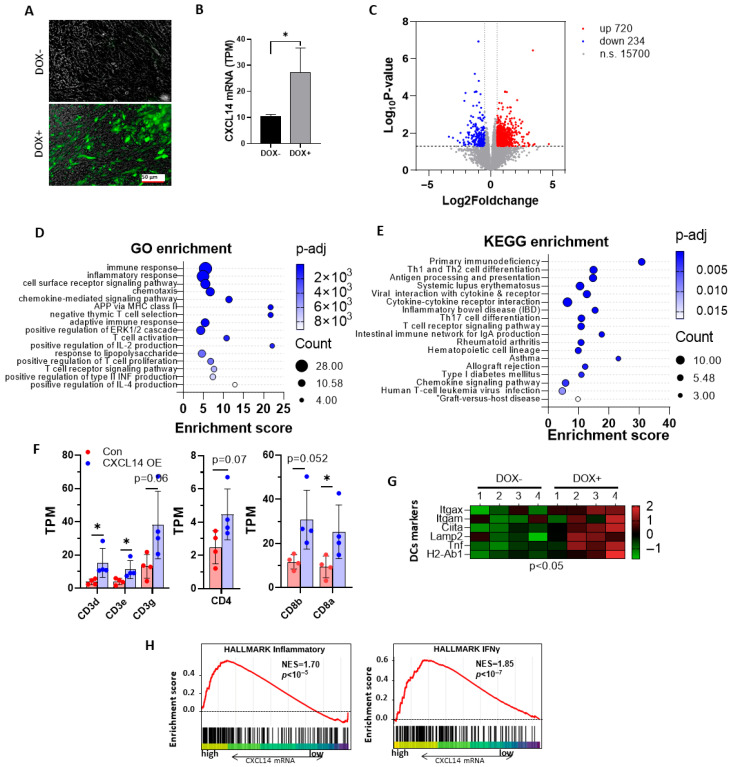
Transcriptome analysis showed that CXCL14 regulates the tumor microenvironment. (**A**) Frozen sections from mice bearing subcutaneous tumors revealed the presence of the reporter gene EGFP, indicating the induction of CXCL14 expression within the tumor. (**B**) RNA-seq data of the expression level of CXCL14 in non-induced and DOX-induced CXCL14 overexpression tumor tissues (*n* = 4), * *p* < 0.05, *t*-test. (**C**) RNA-seq results revealed that there were 720 significantly upregulated genes and 234 downregulated genes in CXCL14 OE MC38 subcutaneous tumors compared to MC38 non-induced tumors. (**D**) The GO analysis results indicate that the gene changes caused by CXCL14 overexpression are mainly concentrated in immune/inflammatory response, chemotaxis, and T cell-related signal transduction. (**E**) The KEGG enrichment analysis results indicate that the gene changes caused by CXCL14 overexpression are mainly concentrated in immune deficiency, Th cell differentiation, and antigen presentation. (**F**) The RNAseq data of T cell marker genes in the subcutaneous tumor with non-induced and DOX-induced CXCL14 overexpression (*n* = 4), * *p* < 0.05, *t*-test. (**G**) The heat map of qPCR data of dendritic cell markers in the subcutaneous tumor with non-induced and DOX-induced CXCL14 overexpression (*n* = 4), *p* < 0.05, *t*-test. (**H**) GSEA of hallmark inflammatory and IFNγ gene signatures was performed to compare CXCL14-overexpressed MC38 cells with non-induced MC38 cells.

MC38-TetOn-CXCL14 cells were used for subcutaneous tumor studies. Upon reaching 1000 mm^3^ tumor volume, mice received DOX-containing drinking water. Frozen tumor sections showed significant EGFP expression (Figure 5A). RNA-seq analysis indicated a CXCL14 upregulation post-DOX induction (Figure 5B). The volcano plot showed CXCL14-induced changes in 720 genes (up) and 234 genes (down), representing 5.7% of total genes (Figure 5C).

GO cluster analysis revealed CXCL14’s impact on immune-related pathways, including immune response, inflammation, T cell activation, and cytokine production (Figure 5D). The regulatory network generated by the enrichment analysis is shown in Figure S9. The KEGG analysis focused on T cell differentiation and antigen presentation (Figure 5E). RNA-seq results showed elevated T cell markers in CXCL14-overexpressing tumors (Figure 5F). Dendritic cell markers were also increased (Figure 5G). GSEA with RNA-seq data demonstrated enrichment of inflammatory pathways and IFNγ-related signatures (Figure 5H).

### 3.6. CXCL14 Mediates T-Cell Infiltration into Subcutaneous Tumors

**Figure 6:**
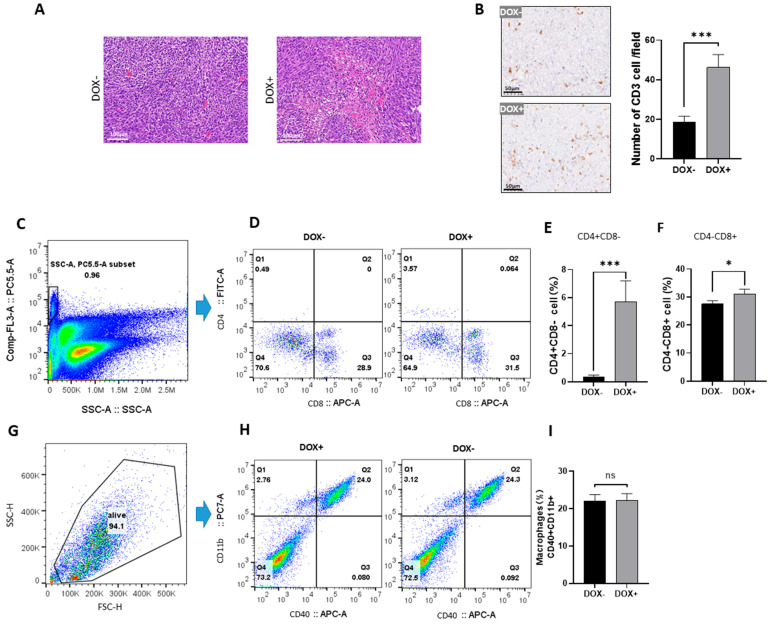
CXCL14 enhances tumor T cell infiltration. (**A**) The H-E staining of the tumor showed that there were more necrotic areas in the tumor overexpressing CXCL14. (**B**) The abundance of CD3 cells was significantly increased in the tumor tissues induced by DOX+ overexpression of CXCL14. Each treatment had 4 biological replicates, and 5 high-power fields were randomly selected from each tumor to count the positive cells. (**C**) The CD3 (PE-Cy5.5) and SSC scatter plot of the tumor cell suspension. The gate in the figure selects CD3-positive T lymphocytes. (**D**) The flow cytometry results of CD4 (FITC) vs. CD8 (APC). The Q1 quadrant represents CD4+CD8-T lymphocytes, and the Q3 quadrant represents CD4-CD8+ T lymphocytes. (**E**) The statistical results of the Q1 quadrant cells in (**D**) show that CD4+CD8- T lymphocytes were significantly increased in the tumor cells induced by DOX overexpression (*p* < 0.001). (**F**) The statistical results of the Q3 quadrant cells in (**D**) showed that CD4-CD8+ T lymphocytes were significantly increased in the tumor cells induced by DOX overexpression (*n* = 4), * *p* < 0.05, *** *p* < 0.001, *t*-test. (**G**) The scatter plot of FSC/SSC of the tumor cell suspension. The gate encloses live cells. (**H**) The scatter plot of CD40/APC and CD11b/PC7 of tumor cells. The Q2 quadrant represents double-positive cells, which are tumor macrophages. Colors indicate the relative cell density in each region of the plot. (**I**) There was no significant change in the content of macrophages in tumor-infiltrating cells (*n* = 4), *t*-test.

We conducted histological studies on subcutaneous tumors and observed that the tumor tissues overexpressing CXCL14 had more necrotic tissue areas (Figure 6A). Immunostaining with the CD3 antibody revealed a significant increase in the number of CD3+-positive cells (Figure 6B). Flow cytometry analysis of total tumor cells revealed that T cells accounted for approximately 1% of the total cells in the tumor tissues (Figure 6C,D). Analysis of CD4 and CD8 cell numbers (Figure 6E) showed that the proportion of CD4 T cells was significantly increased in the tumor overexpressing CXCL14, while the proportion of CD8 T cells was slightly increased (Figure 6F,G). The flow cytometry analysis of macrophage levels revealed that there was no significant increase in the count of CD40 and CD11b double-positive macrophages.

## 4. Discussion

CXCL14 is one of the most highly conserved chemokines, as demonstrated by Huising et al. [8]. It was raised from two gene duplications of the CXC ancestor chemokine gene and has since undergone independent evolution. The Ks/Ka ratio highlights that CXCL14 is subject to the most stringent purifying selection pressure among all CXC chemokines, as depicted in Figure S10, further underscoring its critical functions.

Despite its high conservation, CXCL14 remains enigmatic within the chemokine family. The receptor for this molecule remains unresolved, with ongoing debate centering on whether CXCR4 serves as its natural receptor [29,30,31,32]. Using the β-arrestin assay, Kouzeli et al. conducted a comprehensive investigation into the interactions of CXCL14 with known chemokine receptors and found that CXCL14 is unable to activate any members of the chemokine receptor family, including CXCR4, independently. Instead, it solely participates in signal transduction functions in conjunction with other ligands through synergistic interactions [33].

Chemokines play a pivotal role in modifying the tumor microenvironment. However, given the diverse nature of chemokines as outlined above, the same chemokine ligand can exhibit distinct microenvironment regulatory functions and corresponding prognosis in different tumor types. CXCL14 serves as a prime example of a multifunctional factor. Shabgah et al. and Westrich et al. have reviewed the distinct expression change trends and prognosis of CXCL14 in various organ tumors [34,35].

In CRC, CXCL14 exhibits a tumor-suppressing effect. Previous studies have shown that CXCL14 can inhibit tumor proliferation, migration, and invasion, among other functions in vitro [5,6]. Additionally, bioinformatics research has also revealed the phenomenon of hypermethylation-associated silencing of CXCL14 in CRC, which is associated with poor prognosis [12]. This supports the hypothesis that CXCL14 acts as a tumor suppressor gene in CRC. This study once again demonstrates the inhibitory effect of CXCL14 on colon cancer both in vivo and in vitro.

The results show that the overexpression of CXCL14 is associated with inflammation and IFN-γ, and that the overexpression of CXCL14 inhibits cell proliferation. Although there is no direct evidence that the inhibition of cell proliferation by CXCL14 is mediated by inflammation and IFN-γ, the role of inflammation and IFN-γ in tumor immunity has been widely recognized. For example, IFN-γ has been shown to enhance anti-tumor immune responses in various tumor models by activating immune cells and promoting the immunogenicity of tumor cells [36]. Additionally, inflammatory responses play a dual role in the tumor microenvironment, potentially promoting tumor progression as well as suppressing tumors by activating the immune system [37]. In our experiments, the upregulation of inflammation- and IFN-γ-related genes in tumor tissues overexpressing CXCL14 may indicate that these pathways play a role in tumor growth inhibition. Future studies could further explore the specific mechanisms of these pathways, such as by using IFN-γ-blocking antibodies or anti-inflammatory agents to assess their impact on the growth of tumors overexpressing CXCL14. Such experiments would help to more directly demonstrate the role of inflammation and IFN-γ in CXCL14-mediated tumor suppression.

Mesenchymal–epithelial transition (MET) involves the transition of mesenchymal cells back to the epithelial phenotype, which is a cellular process that reverses EMT. This process leads to enhanced cell–cell adhesion, reduced cell motility, and heightened sensitivity to apoptosis. EMT, a cell migration mechanism during embryonic development, can also activate tumor cells’ abilities to proliferate and invade [38]. Inducing the reverse process of MET has been proposed as a potential new treatment strategy for tumors. CXCL14, one of the earliest chemokines, emerged during the same evolutionary period as the chordate nervous system [8]. Its knockout led to abnormal neuron migration and partial embryonic death, highlighting the crucial role of CXCL14 in embryonic development [39]. We have demonstrated that CXCL14 promotes the MET characteristics of tumor cells in this study.

We previously demonstrated through gene knockout mice that CXCL14 has an important impact on neural migration in the cerebral cortex and embryonic survival rate [40]. Therefore, we believe that CXCL14 is an important gene that regulates EMT in colon cancer, and *CXCL14* gene silencing is one of the switches for tumor cells to enter the EMT stage. The data from this study provide support for the view. Firstly, the overexpression of CXCL14 resulted in a downregulation of 12 MMPs, which are crucial markers of EMT (Figure 3F), consistent with the report of Kuang et al. that CXCL14 negatively regulates MMP2 and MMP9 expression in trophoblast cells [41]. Secondly, through analysis of multiple clinical colon cancer datasets, we found that CXCL14 is negatively associated with key genes involved in EMT, including FN1, CDH2, and the majority of MMPs (Figure 1E,H). These findings suggest that CXCL14 may inhibit tumor metastasis and invasion by promoting the MET process.

Angiogenesis is an important mechanism for suppressing tumor growth. According to the results of transcriptome differential enrichment analysis, CXCL14 can regulate the expression of angiogenesis-related genes. Among them, ADGRG1, an angiogenesis inhibitor gene [42,43], was strongly upregulated by CXCL14 in both in vitro and in vivo models in this experiment (Figure S11), which deserves further attention.

In terms of signal transduction, we have tested the effect of CXCL14 on the MAPK signaling pathway, which is a typical GPCR-mediated intracellular signaling pathway that affects cell motility, growth, and differentiation. The experimental results show that CXCL14 has a significant inhibitory effect on MAPK (Figure 4F). Nevertheless, the precise signal transduction mechanism remains to be further investigated.

Previous studies have demonstrated that CXCL14 regulates the tumor microenvironment by influencing DC cell infiltration in human head and neck cancer and DC cell chemotaxis in vitro [1,44], as well as recruiting NKs in the uterus of mice and NKs in vitro [4,45]. This study found that CXCL14 not only recruits DC and NK cells but also has the function of recruiting and activating T cells, which was first discovered in colon cancer. Parikh et al. found that overexpression of CXCL14 in oral cavity squamous cell carcinoma can recruit T cell infiltration, and that the deletion of T cells eliminates the tumor-suppressing effect of CXCL14 [46], which is consistent with our findings in this study.

In our experiments, the proportions of CD4+ and CD8+ T cells in the tumor tissues of DOX− (non-induced CXCL14 overexpression) mice were relatively low, while these proportions were significantly increased in the tumor tissues of DOX+ (induced CXCL14 overexpression) mice. This is consistent with the expected outcome that CXCL14 promotes T cell infiltration. This difference may reflect the important role of CXCL14 in regulating the tumor immune microenvironment. Although the proportions of CD4+ and CD8+ T cells were lower in DOX− mice, such variations in proportion are not uncommon in the tumor microenvironment, as the tumor microenvironment often suppresses T cell infiltration and activity [47].

CD3+ T cells accounted for approximately 1% of the total cells in the tumor tissue, with CD4+ and CD8+ T cells making up about 30% of the CD3+ T cells. This implies the existence of other CD3+ T cell subsets besides CD4+ and CD8+ T cells. These may include γδ T cells, NKT cells, etc., which may also play important roles in the tumor microenvironment. To gain a more comprehensive understanding of the impact of CXCL14 overexpression on the tumor immune microenvironment, it is necessary to further analyze the functions and proportions of these other CD3+ T cell subsets. This will help us more accurately assess whether CXCL14 overexpression can transform “cold” tumors into “hot” tumors. For instance, γδ T cells and NKT cells also play significant roles in anti-tumor immunity, and their infiltration and activity may be affected by CXCL14 overexpression.

Some of the phenomena revealed in this study are worthy of further investigation. Among them, the conditioned medium of CXCL14 can significantly inhibit the AKT and ERK signaling pathways. However, whether CXCL14 directly acts on a receptor or whether the secondary products generated by the CXCL14 signaling pathway led to this result needs to be studied in detail.

Several limitations of this study should be acknowledged. First, our survival analyses were retrospective and require prospective validation. Second, the subcutaneous tumor model does not fully recapitulate the native colorectal microenvironment. Third, the present study focused primarily on cancer cell-autonomous functions of CXCL14. Whether CXCL14 derived from cancer-associated fibroblasts exerts similar paracrine effects on colon cancer cells, and whether such effects synergize with or differ from cancer-cell-derived CXCL14, warrants further investigation using co-culture systems. Despite these limitations, our findings provide compelling evidence that CXCL14 is a promising therapeutic target for colon cancer.

In summary, our study demonstrates that CXCL14 exerts an inhibitory effect on the development of colon cancer, which is attributed to two functional aspects. Firstly, CXCL14 exhibits the capacity to suppress the proliferation, migration, and metastasis of tumor cells. Secondly, CXCL14 also modulates the immune microenvironment of colon cancer, increasing the levels of NKs, DCs, and T cells, and converting the tumor from a “cold” to a “hot” phenotype. These findings suggest that CXCL14 could serve as a potential target for immunotherapy in colon cancer.

## Data Availability

Data are available in a public, open-access repository. The MC38 cell RNA-seq data are available through GEO access number GSE256328 (https://www.ncbi.nlm.nih.gov/geo/query/acc.cgi?acc=GSE256328, (accessed on 1 February 2025)). The subcutaneous tumor RNA-seq data are available through GEO access number GSE256330 (https://www.ncbi.nlm.nih.gov/geo/query/acc.cgi?acc=GSE256330, (accessed on 1 February 2025)).

## Supplementary Materials

The following supporting information can be downloaded at https://www.mdpi.com/article/10.3390/cells15100860/s1. Figure S1. The correlations between expressions of CXCL14 and EMT markers. Figure S2. The correlations between expressions of CXCL14 and TK1. Figure S3. CXCL14 expression predicts survival outcomes in colorectal cancer. Figure S4. The correlation coefficients of CXCL14 and immune cell biomarkers. Figure S5. Structure of CXCL14 inducible plasmid and expression. Figure S6. DOX does not affect the proliferation and invasion of MC38 cells. Figure S7. Transient transfection of CXCL14 inhibits MC38 cell invasion. Figure S8. The overexpression of CXCL14 inhibits the expression of EMT-related genes in HCT cells. Figure S9. GO-dotplot of differentially expressed genes in subcutaneous tumors Figure S10. Ka/Ks ratio of CXC chemokine and receptor family. Figure S11. CXCL14 upregulates ADGRG1 mRNA in CRC and normal colon. Table S1. Primers for qPCR. References [48,49] are cited in Supplementary Materials.

## Abbreviations

CRC: colorectal carcinoma; DCs: dendritic cells; DEGS: differently expressed genes; DOX: doxycycline; EMT: epithelial–mesenchymal transition; GO: Gene Ontology; GSEA: Gene Set Enrichment Analysis; GTex: Genotype-Tissue Expression; KEGG: Kyoto Encyclopedia of Genes and Genomes; MMPs: matrix metalloproteinases; NES: Normalized Enrichment Score; NKs: natural killer cells; Treg: regulatory T cells; WB: Western blot.
