# Supplementary material for: CXCL14 Inhibits Colon Cancer Progression by Modulating Tumor Cell Invasion and Immune Microenvironment
Source: Cells. 2026 May 8;15(10):860. doi: 10.3390/cells15100860 (PMC13204761; doi:10.3390/cells15100860)
Supplement: Supplementary file 1 [file cells-15-00860-s001.zip › cells-4248955-supplementary.pdf]

CXCL14 suppresses the progression of colon cancer by regulating tumor epithelial-mesenchymal transition and tumor microenvironment

Yinjie Zhang[1,2], Siyi Wang[1], Yuchen Niu[1], Yanjing Wang[1,3], Buyong Ma[1,3], Jingjing Li[1,3,*]

1 School of Pharmacy, Shanghai Jiao Tong University, 800 Dongchuan Road, Shanghai 200240, China; zhyj@sxu.edu.cn (Y.Z.); wangsiyi_sjtu@163.com (S.W.); nyc123@sjtu.edu.cn (Y.N.);
wangyanjing@sjtu.edu.cn (Y.W.); mabuyong@sjtu.edu.cn (B.M.)
2 Biomedical and Health Laboratory in Shanxi Province, School of Life Science, Shanxi University, Tai-yuan, 030006, Shanxi, China
3 Engineering Research Center of Cell and Therapeutic Antibody, Ministry of Education, 800 Dongchuan Road, Shanghai 200240, China
* Correspondence: lijj@sjtu.edu.cn

Supplementary Materials

Fig. S1. The correlations between expressions of CXCL14 and EMT markers.
Fig. S2. The correlations between expressions of CXCL14 and TK1.
Fig. S3. CXCL14 expression predicts survival outcomes in colorectal cancer.
Fig. S4. The correlation coefficients of CXCL14 and immune cell biomarkers.
Fig. S5. Structure of CXCL14 inducible plasmid and expression.
Fig. S6. DOX does not affect the proliferation and invasion of MC38 cells.
Fig. S7. Transient transfection of CXCL14 inhibits MC38 cell invasion.
Fig. S8. The overexpression of CXCL14 inhibits the expression of EMT-related genes in HCT cells.
Fig. S9. GO-dotplot of differentially expressed genes in subcutaneous tumors
Fig. S10. Ka/Ks ratio of CXC chemokine and receptor family.
Fig. S11. CXCL14 upregulates ADGRG1 mRNA in CRC and normal colon.
Table. S1. Primers for qPCR

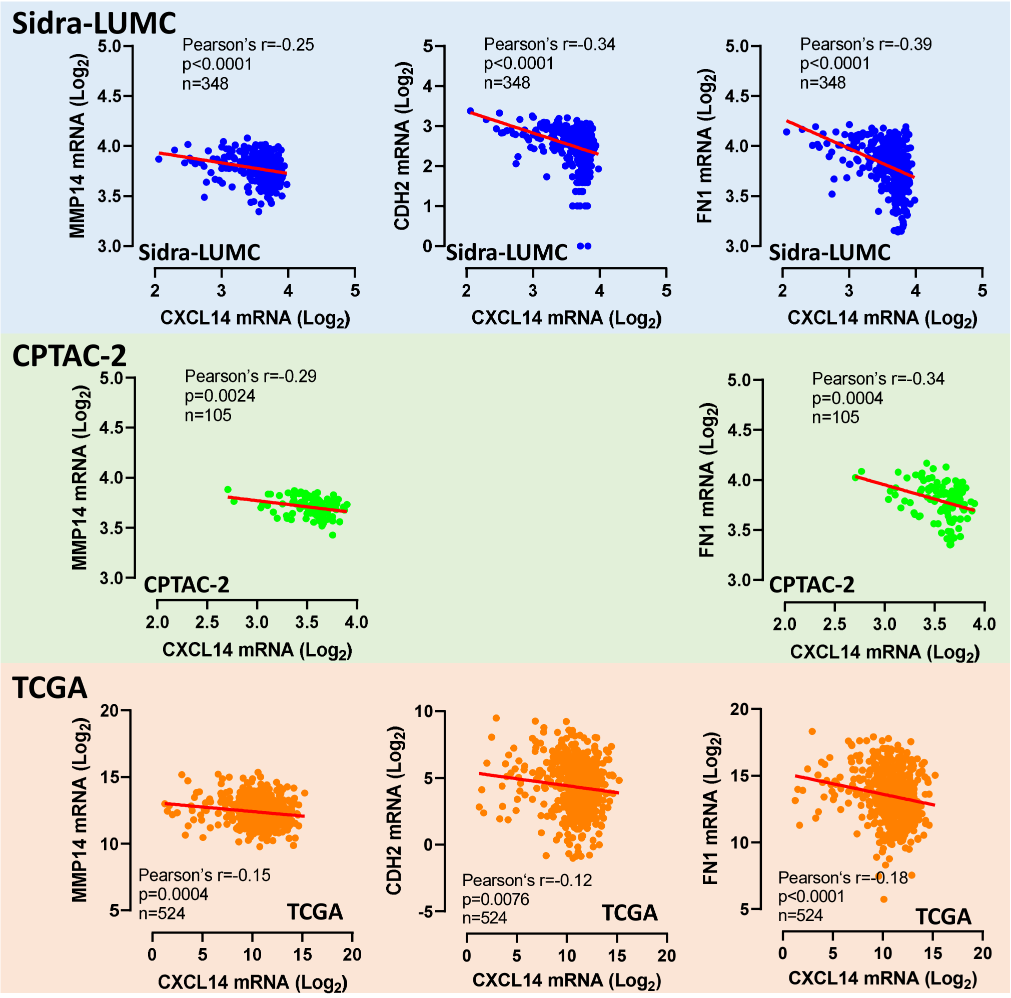

Supplementary Figure 1. The correlations between expressions of CXCL14 and EMT markers. The expression data were derived from three databases: Sidra-LUMC, CPTAC-2, and TCGA. The significance of correlation was obtained through the Pearson test. The CPTAC-2 database did not contain data for CDH2.

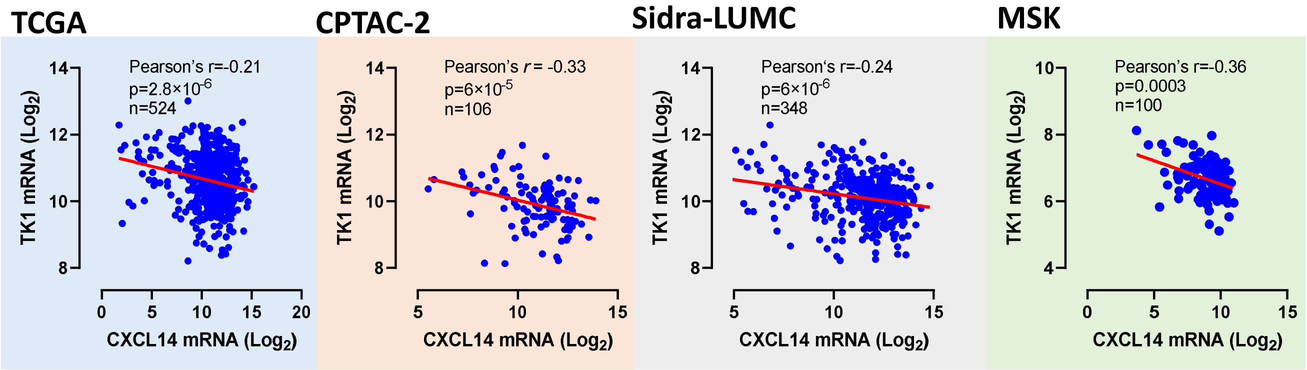

Supplementary Figure S2. The correlations between expressions of CXCL14 and TK1. The correlation between the mRNA levels of TK1 and CXCL14 was examined in four different colorectal cancer databases. The correlation was found to be significantly negative in all of them.

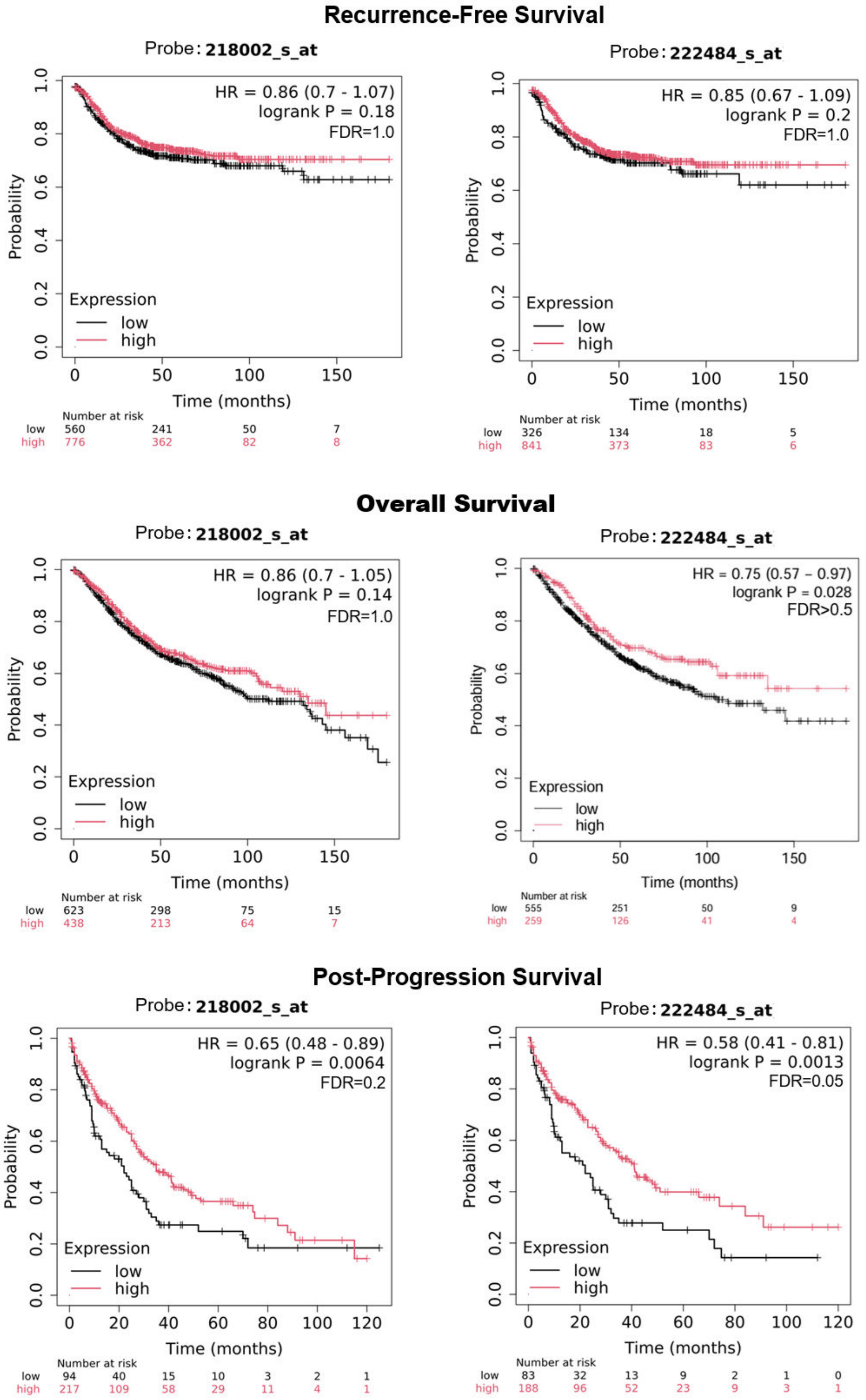

Supplementary Figure S3. CXCL14 expression predicts survival outcomes in colorectal cancer
Prognostic impact of CXCL14 expression in tumor tissues on different survival outcomes in patients with colorectal cancer (CRC). CXCL14 expression was evaluated by two probes (218002_s_at, 222484_s_at).

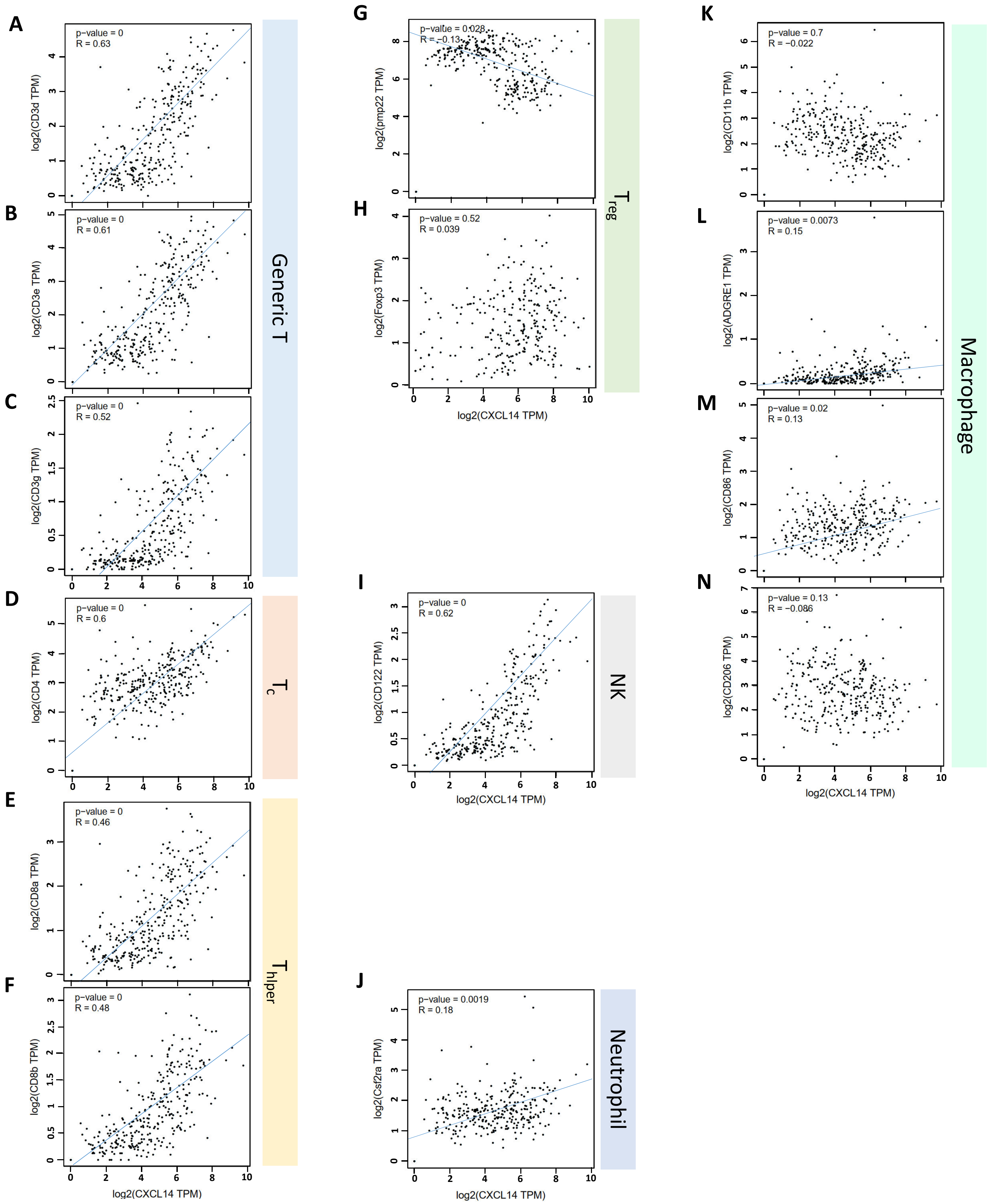

Supplementary Figure S4. The correlation coefficients of CXCL14 and immune cell biomarkers

(A-C). CXCL14 in colon tissue (Sigmoid and Transverse) is expressed strongly correlated ($p<10^{8}$, R>0.5) with T cell marker CD3d, CD3e, and CD3f; **(D)**. CXCL14 in colon tissue (Sigmoid and Transverse) is expressed correlated with Tc biomarker CD4 ($p<10^{8}$, R>0.4); **(E-F).** CXCL14 expression in colon tissue is strongly correlated with T help biomarker CD8a, CD8b($p<10^{8}$, R>0.4); **(G-H)**. CXCL14 shows no or negative correlation with T_{reg} biomarker Pmp22 and Foxp3. **(I)**. CXCL14 shows significant positive correlation with NK biomarker CD122. **(J)**. CXCL14 shows weak positive correlation with Neu biomarker Csf2ra (r=0.18). **(K-N).** CXCL14 shows weak or no correlation with Macrophage biomarker CD11b, ADGRE1(r=0.15), CD86(r=0.13) and CD206

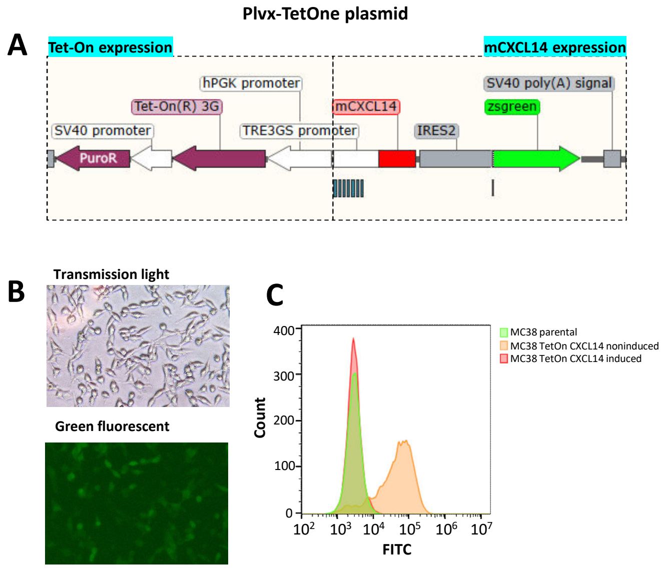

Supplementary Figure S5. Structure of CXCL14 inducible plasmid and expression.

(A)The structure of the inducible CXCL14 expression plasmid. The plasmid is derived from the tetracycline-inducible expression plasmid system. The plasmid constitutively expresses the Tet-On(R)3G protein, which has promoter activity in the presence of tetracycline derivatives and can bind to the TRE3GS promoter to activate the mRNA transcription of downstream genes. The transcriptional unit in the plasmid includes the mouse CXCL14 gene, the IRES2 sequence, and the zsgreen gene. The zsgreen gene serves as a fluorescent reporter gene to indicate the expression of mCXCL14 gene.

(B)After inducing MC38-TetOn-CXCL14 cells with 1ug/mL DOX for 48h, fluorescence microscopy images indicate that the reporter gene zsgreen is clearly expressed in the cells.

(C)Flow cytometry was used to detect the expression of EGFP in MC38-TetOn-CXCL14 cells before and after induction. The cells before induction were similar to parental MC38 cells, while the signal was significantly increased after induction.

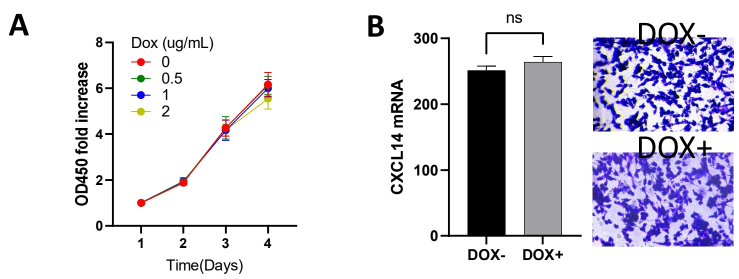

Supplementary Figure S6. DOX does not affect the proliferation and invasion of MC38 cells.

(A) The growth rate of MC38 cells cultured with 0.5~2ug/ml doxycycline is not affected.

(B) The tumor invasion ability of MC38 cells cultured with 1 ug/ml doxycycline is not significantly changed.

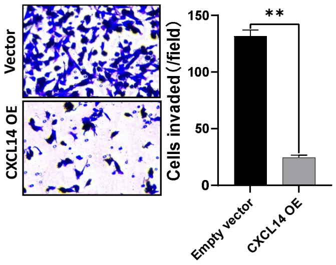

Supplementary Figure S7. Transient transfection of CXCL14 inhibit MC38 cell invasion. The transient transfection of MC38 cells with the plasmid pcDNA-CXCL14 for overexpression of CXCL14 can significantly inhibit the invasive ability of the cells (n=3) . **$p<0.01$, t-test.

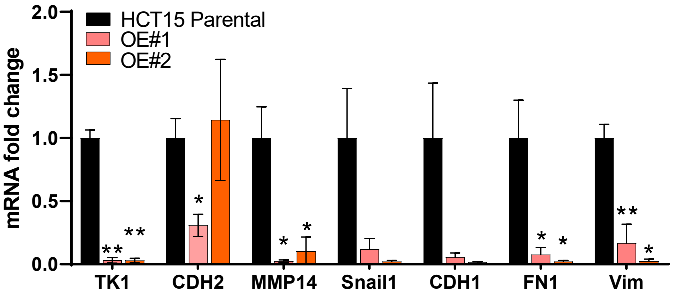

Supplementary Figure S8. The overexpression of CXCL14 inhibits the expression of EMT-related genes in HCT cells. Q-PCR analysis of the expression levels of pertinent genes in HCT15 cell lines overexpressing CXCL14, it was observed that the overexpression of CXCL14 led to significant inhibition of tumor proliferation marker (TK1) and tumor EMT markers (n=2). *p<0.05, **p<0.01, t-test.

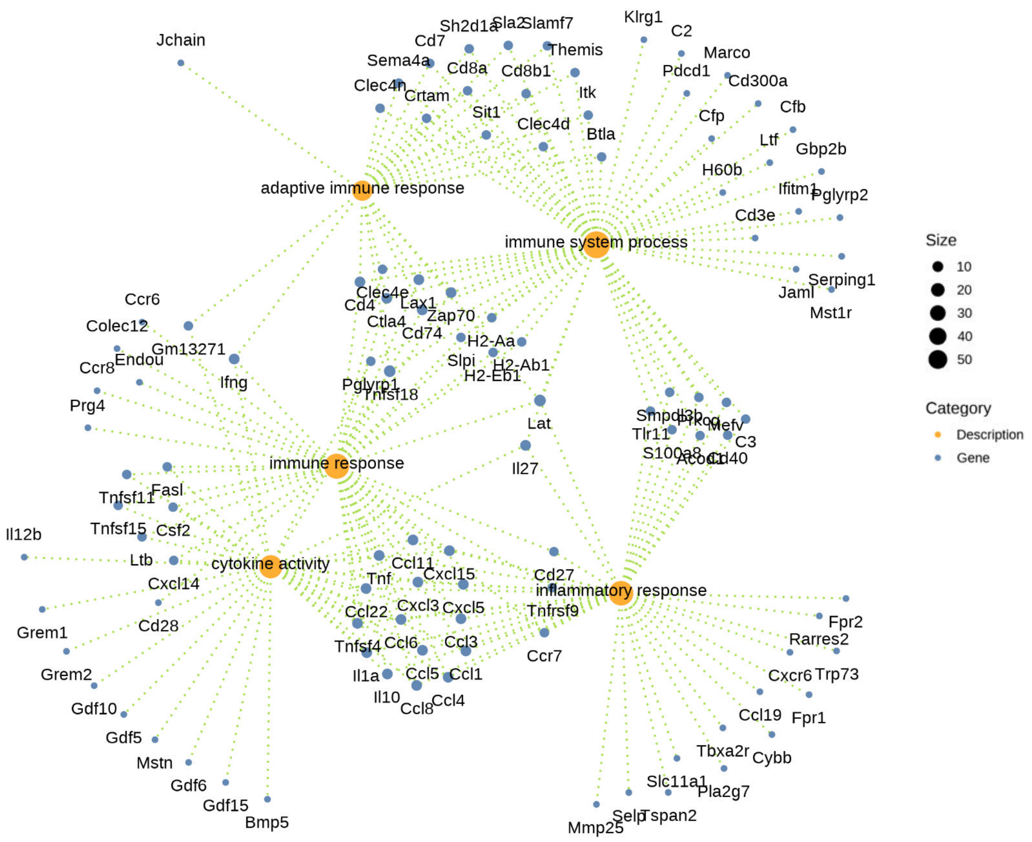

Supplementary Figure S9. GO-dotplot of differentially expressed genes in subcutaneous tumors

A

Human genes	mice genes	NG	GY
**CXC Ligand subfamily**			
Cxcl1	*cxcl3*	0.368	0.364
Cxcl2	*cxcl1*	0.373	0.342
Cxcl3	*cxcl2*	0.251	0.251
Cxcl7	*cxcl7*	0.541	0.495
Cxcl4	*cxcl4*	0.246	0.247
Cxcl6	*cxcl5*	0.275	0.348
Cxcl9	*cxcl9*	0.296	0.251
Cxcl10	*cxcl10*	0.195	0.236
Cxcl11	*cxcl11*	0.159	0.060
Cxcl13	*cxcl13*	0.777	1.079
Cxcl14	*cxcl14*	0.044	0.019
Cxcl12	*cxcl12*	0.048	0.033
Cxcl16	*cxcl16*	0.610	0.736
**CXC receptor subfamily**			
Cxcr1	*cxcr1*	0.581	0.492
Cxcr2	*cxcr2*	0.764	0.770
Cxcr3	*cxcr3*	0.186	0.177
Cxcr4	*cxcr4*	0.120	0.082
Cxcr5	*cxcr5*	0.354	0.253
Cxcr6	*cxcr6*	0.245	0.196
**House keeping gene**			
Gapdh	*gapdh*	0.077	0.058
Flnb	*flnb*	0.034	0.034

B

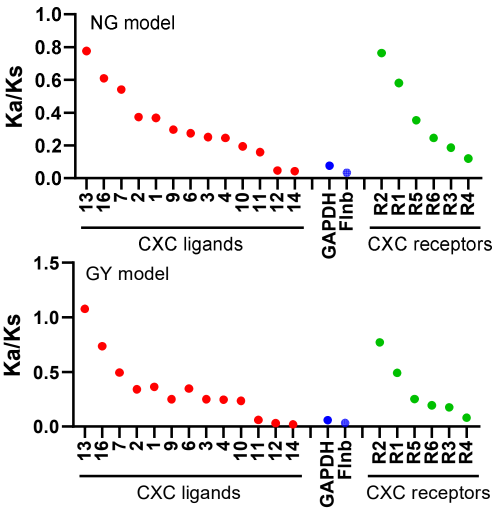

Supplementary Figure S10. Ka/Ks ratio of CXC chemokine and receptor family.(A) The left column shows the Ka/Ks of each gene calculated using software KaKs_calculator Version 1.2 (http://evolution.genomics.org.cn / software.htm) with homologous genes of human and mouse by NG, an approximate algorithms (Nei M. and Gojobori T., Mol Biol Evol 1986;3:418-26.), and GY, a Maximum-Likelihood Methods (Goldman N., et al. Mol Biol Evol, 1994, 11: 725-736). Ka/Ks is calculated in mature peptide of chemokines, ectodomains of receptors, and *Gapdh* and *Flnb* of human and mouse as references. **(B)** Scatter diagram of Ka/Ks of ligand and receptor family. The results of two algorithems insistently indicated that *Cxcl14* has the lowest Ka/Ks ratio in ligand family, the value even lower than that of housekeeping genes *Gapdh* and *Flnb* in GY model. In the receptor family *CXCR4* the receptor gene of CXCL12 has the lowest Ka/Ks ratio, on the contrary, *CXCR2* exhibited the highest Ka/Ks, indicating a coevolution mode between CXC ligand and receptor.

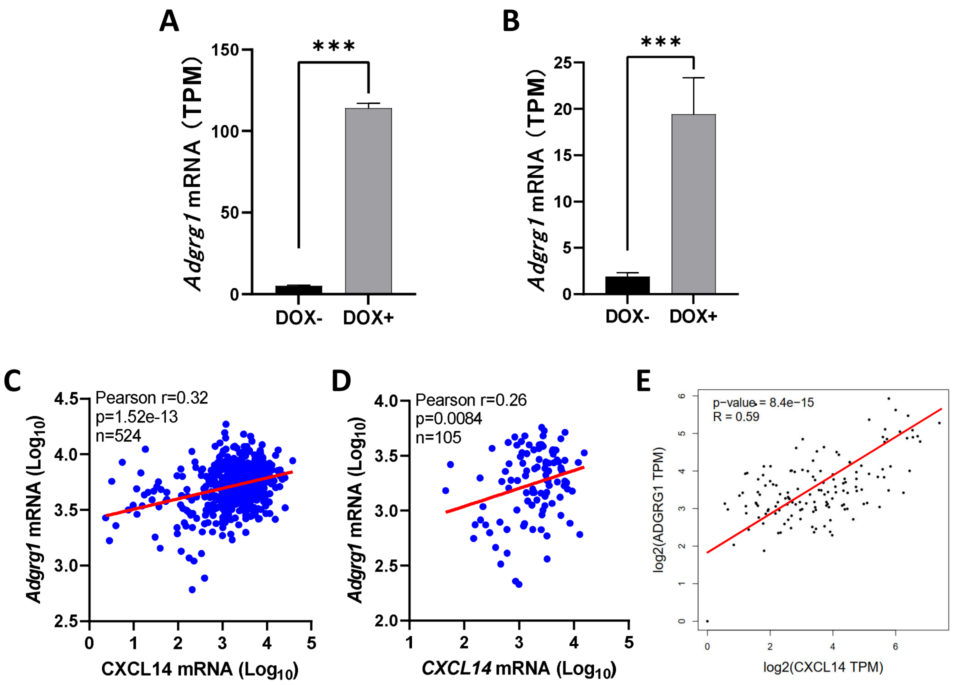

Supplementary Figure S11. CXCL14 upregulates ADGRG1 mRNA in colorectal carcinoma and normal colon

(A) The overexpressed CXCL14 (induced by DOX) significantly enhances the expression of ADGRG1 in MC38 cells grown in vitro. **(B)** The overexpression of CXCL14 (induced by DOX) promotes the expression of ADGRG1 in MC38 cells grown as subcutaneous tumors. **(C)** and **(D)** The database TCGA and CPTAC colon cancer demonstrate a significant positive correlation between CXCL14 and ADGRG1. **(E)** In the GTEx database, a significant positive correlation was observed between CXCL14 and ADGRG1 in normal colon tissue (n=136).

Supplementary Table S1. Primers for qPCR

·Primer	Sequence (5'-3')	target
mCXCL14-q4s	GCACTGCCTGCACCCTAAG	Endogenous mouse CXCL14 mRNA
mCXCL14-q4a	CTTCGTAGACCCTGCGCTTC	
mCXCL14-Q3s	CAGAGCACCAAACGCTTCATC	Plasmids derived mouse CXCL14 mRNA
mCXCL14-Q3a	TTCTTTCCATGATCGTCCACCC	
hCXCL14-qs	AAGCCAAAGTACCCGCACTG	Endogenous mRNA or plasmids derived human CXCL14 mRNA
hCXCL14-qa	GACCTCGGTACCTGGACACG	
mMmp14-qs	TTCCAATGATCCCTCCGCCA	Endogenous mouse Mmp14
mMmp14-qa	GACCCTGACTTGCTTCCATAAA	
mFn1-qs	GACGCCGTTCCAGGAGAGTT	Endogenous mouse Fn1
mFn1-qa	AGTCAGAGTCGCACTGGTAGA	
mTK1-qs	AGCAACAGCTTCTCCACACA	Endogenous mouse TK1
mTK1-qa	CAAGGACTCCTGGGTCACATC	
mCDH2-qs	TTCTGGCGGCCTTGCTT	Endogenous mouse CDH2
mCDH2-qa	CGGTAAGACTGCGCTGTAAA	
mCDH1-qs	AACGCTCCTGTCTTCAACCC	Endogenous mouse CDH1
mCDH1-qa	GGTCACTTTGAGTGTGGCGA	
mTwist1-qs	TTCACAAGAATCAGGGCGTG	Endogenous mouse Twist1
mTwist1-qa	CTGCCCCTCTGGGAATCTCT	
mVim-qs	AGCACCCTGCAGTCATTCAG	Endogenous mouse Vim
mVim-qa	TCCACTTTCCGTTCAAGGTCA	
mSnai1-qs	GTCCAGCTGTAACCATGCCT	Endogenous mouse Snail1
mSnai1-qa	TGTCACCAGGACAAATGGGG	
mSox2-qs	GGAGGAGAGCGCCTGTTTTT	Endogenous mouse Sox2
mSox2-qa	CTGGCGGAGAATAGTTGGGG	
mOcln-qs	GAACTGTGGATTGGCAGCG	Endogenous mouse Ocln
mOcln-qa	AGCAAAATGTCCAGGCTCCC	
mMmp9-qs	GAGTTCTCTGGTGTGCCCTG	Endogenous mouse Mmp9
mMmp9-qa	TTGGAAACTCACACGCCAGA	
mMmp2-qs	ACGATGATGACCGGAAGTGG	Endogenous mouse Mmp2
mMmp2-qa	GTCCTGAGAGTGTTCCAGCC	
mKrt10-qs	ACGAGAAGCATGGCAACTCA	Endogenous mouse Krt10
mKrtq0-qa	TTGTCAGGGTGAGGATCTGC	
mFas-qs	CCATGCACAGAAGGGAAGGA	Endogenous mouse Fas
mFas-qa	GGGTGCAGTTTGTTTCCACC	
